# On-demand continuous-variable quantum entanglement source for integrated circuits

**DOI:** 10.1515/nanoph-2022-0555

**Published:** 2023-01-18

**Authors:** Mehmet Günay, Priyam Das, Emre Yüce, Emre Ozan Polat, Alpan Bek, Mehmet Emre Tasgin

**Affiliations:** Department of Nanoscience and Nanotechnology, Faculty of Arts and Science, Mehmet Akif Ersoy University, 15030 Burdur, Türkiye; Department of Physics, Bankura Sammilani College, Kenduadihi, Bankura, WB 722101, India; Department of Physics, Middle East Technical University, 06100 Ankara, Türkiye; Faculty of Engineering and Natural Sciences, Kadir Has University, Cibali, Istanbul 34083, Türkiye; Institute of Nuclear Sciences, Hacettepe University, 06800 Ankara, Türkiye

**Keywords:** Fano resonances, quantum integrated circuits, quantum optics, voltage control

## Abstract

Integration of devices generating non-classical states (such as entanglement) into photonic circuits is one of the major goals in achieving integrated quantum circuits (IQCs). This is demonstrated successfully in recent decades. Controlling the non-classicality generation in these micron-scale devices is also crucial for the robust operation of the IQCs. Here, we propose a micron-scale quantum entanglement device whose nonlinearity (so the generated non-classicality) can be tuned by several orders of magnitude via an *applied voltage* without altering the linear response. Quantum emitters (QEs), whose level-spacing can be tuned by voltage, are embedded into the hotspot of a metal nanostructure (MNS). QE-MNS coupling introduces a Fano resonance in the “nonlinear response”. Nonlinearity, already enhanced extremely due to localization, can be controlled by the QEs’ level-spacing. Nonlinearity can either be suppressed or be further enhanced by several orders. Fano resonance takes place in a relatively narrow frequency window so that ∼meV voltage-tunability for QEs becomes sufficient for a *continuous* turning on/off of the non-classicality. This provides as much as 5 orders of magnitude modulation depths.

## 1 Introduction

Quantum optics has been revolutionizing computational power that led to recent demonstration of quantum advantage [1, 2]. This exciting development accompanied by quantum networks [3], utilizing quantum teleportation [4, 5], will surely shape the future. In quantum computers, quantum interconnects replace the classical ones which limit the operation frequencies [6] of conventional computers. Quantum interconnects enable nonlocal quantum operations which make quantum modular architecture possible [7], [8], [9]. While these exciting demonstrations in quantum optics are encouraging, the field is facing major challenges in developing scalable integration of components and most importantly on-demand quantum sources for diminishing errors in computation. The determinism —provided by the on-demand source— brings the advantage that the gate operations can be carried out more accurately since the generation and arrival time of photons are known. If the source is not deterministic, the gate operations would be probabilistic in nature.

The profound efforts on the scalable integration of quantum optics —integrated quantum circuits— which aim to enable operation of quantum computation and quantum communication on a single medium retains a great excitement [10, 11]. Single-photon sources [12], [13], [14], [15] and/or continuous-variable (CV) sources are required to implement a scalable quantum computation scheme. Remarkably, entanglement swapping between discrete-variable and CV optical system indicate a connected nature of the two systems [16]. The latter, however, has its own advantages such as high efficiency state characterization and unconditional state manipulation [17, 18]. CV computation scheme is also compatible with the already existing integrated optical architectures [19].

Integration of a scalable quantum source necessitates the controlled generation and manipulation of entangled and/or squeezed light at much smaller dimensions. This need stimulated research efforts to be focused on generation of quantum states at the micrometer scale, an example of which is the production of the quadrature-squeezed states on silicon nitride chips [19] among other demonstrations of CV entanglement [17, 20], [21], [22], [23], [24]. Nonlinear interactions on chip are the key for generating an entangled CV source. Nonlinear frequency conversion rate is either fixed [18]; or it can be controlled by auxiliary light [25], by tuning the resonances [26] and optical filters [27], by voltage-controlled preparation of two-photon states [28], and by adjusting phase-matching condition [29]. The control on the production of quantum non-classicality in a circuit will provide the key control on a quantum source which can be integrated on a chip.

In this paper, we study an integrable micron-scale entangler where non-classicality generation can not only be switched on/off, but also *tuned continuously* by an applied voltage. The entanglement (non-classicality) switch is based on the Fano-control of nonlinear response of a metal nanostructure (MNS). Furthermore, thanks to Fano-control that the dynamic range of the nonlinear conversion can span as large as 5 orders of magnitude, whereas the linear response remains unaltered. A quantum emitter (QE) is positioned at the hot spot of the MNS and creates the Fano resonance in the nonlinear response. The level-spacing of the QE can be tuned via an applied voltage [26, 30], [31], [32], [33], which also controls the rate of the nonlinear conversion —thus, the non-classicality of the system. Here, as an example, we work the squeezing and entanglement generation at the fundamental frequency (*ω*) of a Fano-controlled second harmonic generation (SHG) process. However, such a control can be achieved also in other nonlinear processes [34, 35].

Fano resonances appear at relatively sharp frequency bands [36, 37]. This feature may be disadvantageous in achieving broadband nonlinearity enhancements in MNSs. Here, however, we turn the sharpness of the Fano resonances into an advantage, because a smaller voltage tuning for the QE level-spacing (*ω*_QE_), ∼meV [26, 30], [31], [32], comes to be sufficient for turning on and off the non-classicality.

**Figure 1:**
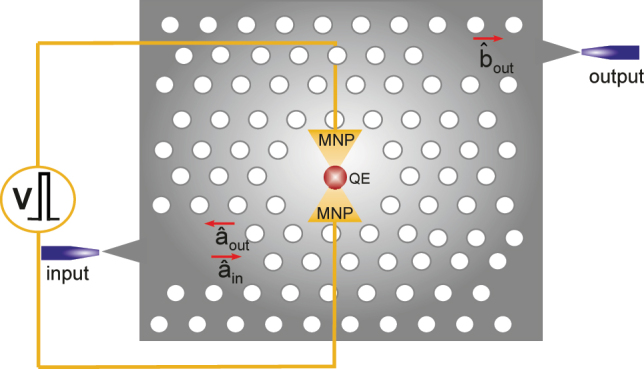
Micron-scale, voltage-tunable integrated entanglement device. Nonlinearity of the MNS is already extremely enhanced due to localization at the hotspot [41]. QE(s) positioned to the hotspot induces a Fano resonance which can suppress (turn off) the localization-enhanced nonlinearity by several orders at *ω*_QE_ = 2*ω* or enhance it 10–100 times at around *ω*_QE_ ≈ 2.002*ω*. Level-spacing (*ω*_QE_) is tuned by an applied voltage [26, 30], [31], [32], [33]. ${\hat{a}}_{\text{in}}$ is the input field (integrated laser), ${\hat{a}}_{\text{out}}$ and ${\hat{b}}_{\text{out}}$ are the output fields whose entanglement (Figure 4a) and non-classicality (Figure 4b) are investigated.

**Figure 2:**
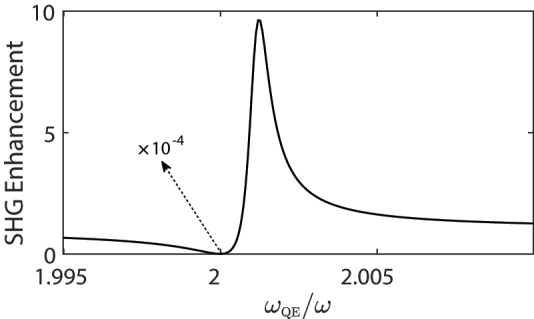
Fano-enhancement of the SHG, which multiplies the localization enhancement [41], for different *ω*_QE_. SHG enhancement is tuned between ×10^−4^ and ×10 by adjusting the QE level-spacing *ω*_QE_ via an applied voltage.

We consider a micron-sized photonic crystal cavity [38] into which a MNS, e.g., a bow-tie antenna, is embedded, see Figure 1. The QE(s) [39], whose level-spacing is voltage-tuned [32], is positioned at a few-nm-sized hotspot in the gap. A Fano resonance appears due to the MNS-QE coupling, see Figure 2. When *ω*_QE_ is tuned to 2*ω*, the SHG process (so the non-classicality generation) is suppressed by 4 orders of magnitude, i.e., multiplied by 10^−4^ [34, 35, 40]. In contrast, when the *ω*_QE_ is tuned to ≈2.002ω , the SHG is enhanced 10 times. We remark that the localization (hotspot) already enhances the SHG, for instance, by 10^6^ times [41]. The pronounced Fano-suppression (enhancement) factors, 10^−4^ and 10, multiplies the 10^6^ localization enhancement [41, 42]. The cavity is pumped by an integrated microlaser [14, 43] on the left hand side.

Here, we show that the SHG process taking place inside the cavity (entanglement device) generates two kinds of non-classsicality. (i) The two waves emitted from the cavity in the opposite directions ( ${\hat{a}}_{\text{out}}$ and ${\hat{b}}_{\text{out}}$ in Figure 1), at the fundamental frequency *ω*, are entangled, see Figure 4a. (ii) The transmitted light ${\hat{b}}_{\text{out}}$ is single-mode non-classical, see Figure 4b. Thus, one can either (i) use the two entangled light beams or (ii) create entanglement (on the right hand side) from the non-classicality inherited in ${\hat{b}}_{\text{out}}$ using an integrated beam-splitter (BS) [44, 45]. The output port guides/transfers [46] the generated squeezed light $\left({\hat{b}}_{\text{out}}\right)$ to other components of the integrated circuit. A BS can be used externally or integrated at the output waveguide inside the photonic crystal. This enables the creation of entanglement in the photonic chip from the squeezed light $\left({\hat{b}}_{\text{out}}\right)$ . (We note in advance that we use the BS only to convert the squeezed light ${\hat{b}}_{\text{out}}$ into two-mode entanglement at the output of the BS. This is because, the two output modes of a BS are entangled if one of the two input modes is non-classical, in particular if it is squeezed [47]. That is, we do not use the BS for homodyne-detection here.) Beyond the generation of non-classicality [48, 49], the most important thing our device can provide is the continuous tunability of the quantum non-classicality (by several orders of magnitude) via an applied voltage with an unaltered linear response.

## 2 Control of quantumness by voltage

### 2.1 Cavity system and Hamiltonian

**Figure 3:**
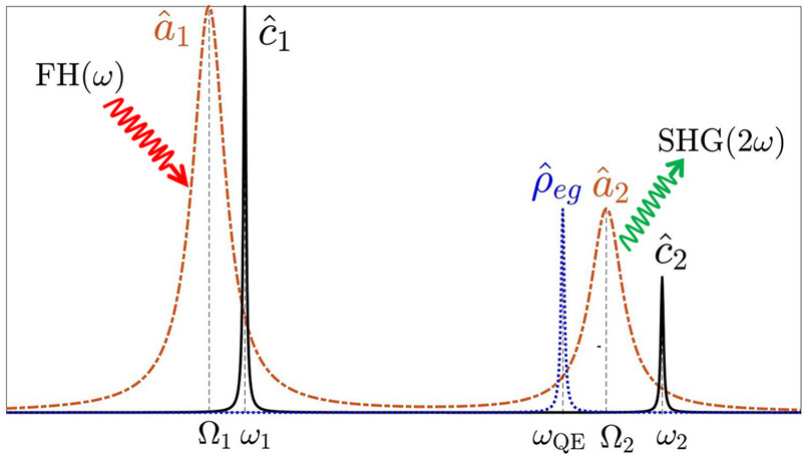
Resonances of the plasmonic $\left({\hat{a}}_{1,2}\right)$ and cavity $\left({\hat{c}}_{1,2}\right)$ modes are depicted by Ω_1,2_ and *ω*_1,2_, respectively. SHG conversion is carried out over the plasmon modes ${\hat{a}}_{1,2}$ due to strong localization [51]. Both first harmonic (FH) and second harmonic (SH) fields are localized in plasmon modes. The resonance of the QE (depicted by *ρ*_*eg*_) is chosen around the ${\hat{a}}_{2}$ plasmon mode —couples to it. The conversion (enhancement) dynamics is given in the text in details.

Dynamics of the voltage-controlled entanglement device can be described as follows. An integrated laser of frequency *ω* excites the fundamental cavity mode $\left({\hat{c}}_{1}\right)$ on the left hand side, ${\hat{H}}_{L}=\hbar \left(\varepsilon_{L}{\hat{c}}_{1}^{†}{\text{e}}^{-\text{i}\omega t}+H.c.\right)$ . The cavity mode couples with the first (lower-energy) plasmon mode ( ${\hat{a}}_{1}$ , resonance Ω_1_) of the bow-tie MNS, ${\hat{H}}_{1}=\hbar g_{1}{\hat{a}}_{1}^{†}{\hat{c}}_{1}+H.c.$ . The relative positions of the resonances are given in Figure 3. The ${\hat{a}}_{1}$ plasmon excitation is localized within the nm-sized hotspot located at the gap between the two metal nanoparticles. Orders of magnitude enhanced electromagnetic field (*ω*), at the hotspot yield the SHG process [50]. Two localized excitations (*ω*) in the ${\hat{a}}_{1}$ plasmon mode combine to produce a single 2*ω* plasmon in the second (higher energy) plasmon mode ${\hat{a}}_{2}$ , ${\hat{H}}_{SHG}=\hbar \chi^{\left(2\right)}{\hat{a}}_{2}^{†}{\hat{a}}_{1}{\hat{a}}_{1}+H.c$ . The hotspot of the ${\hat{a}}_{2}$ mode, of resonance Ω_2_, is also at the center (gap). The SHG conversion takes place over the plasmons [51] because overlap integral for this process is extremely large due to the localization [42, 52]. As both incident (*ω*) and converted (2*ω*) fields are localized, the SHG process can be enhanced as large as 10^6^ times —localization enhancement [41]. The level-spacing of the QE (*ω*_QE_) is around the second harmonic (SH) frequency 2*ω* and the resonance Ω_2_ of the second plasmon mode ${\hat{a}}_{2}$ , so that it is off-resonant to the fundamental frequency. The localized ${\hat{a}}_{2}$ plasmon mode couples with the QE(s) ( ${\hat{H}}_{2}=\hbar f|e〉〈g|{\hat{a}}_{2}+H.c$ .) which introduces a Fano resonance in the SH conversion, see Figure 2. The SHG process can be controlled by the level-spacing of the QE(s), *ω*_QE_ which is tuned by an applied voltage [26, 30], [31], [32], [33]. When voltage tunes the QE(s) to *ω*_QE_ = 2*ω*, the localization enhanced (e.g., about 10^6^ times) SHG is suppressed 10^−4^ times. That is, the switch turns the SHG off. When *ω*_QE_ ≈ 2.002*ω*, this time the localization enhanced SHG is further multiplied by a factor of 10.

In our device, non-classicality (squeezing) is generated at the nonlinear process taking place at the hot spot (plasmon modes) and it is transformed into the cavity output as follows. The SHG process ( ${\hat{a}}_{2}^{†}{\hat{a}}_{1}{\hat{a}}_{1}+H.c$ .) generates the squeezing (non-classicality) in the ${\hat{a}}_{1}$ plasmon mode [53]. The non-classicality of the ${\hat{a}}_{1}$ mode is transferred back to the fundamental cavity mode ${\hat{c}}_{1}$ via the BS interaction, as ( ${\hat{c}}_{1}^{†}{\hat{a}}_{1}+H.c$ ). A BS interaction is known for transferring the squeezing in one mode to the second one [49]. Thus, non-classicality is introduced in the ${\hat{c}}_{1}$ mode. Similarly, the cavity mode couples to the output mode ${\hat{b}}_{\text{out}}$ , again, via a BS type interaction $\left({\hat{b}}_{\text{out}}{\hat{c}}_{1}+H.c.\right)$ [54, 55]. Thus, the squeezing is now transferred into the ${\hat{b}}_{\text{out}}$ mode which makes the output of the device (propagating along the right-hand-side direction into the silicon-based waveguide) non-classical, see Figure 1. This non-classical light can be used to create two-mode (or multimode [56, 57]) entanglement at an integrated beam-splitter [44, 45, 47] placed on the right-hand-side. It is worth noting that a BS also generates entanglement between the two modes if one of the modes possesses non-classicality. (Not all of the total non-classicality can be converted into entanglement in BS like interactions, but some single-mode non-classicality remains within the transferred mode [58].) By tuning the nonlinear process taking place at the hot spot (within the plasmon modes) we tune the plasmonic non-classicality ( ${\hat{a}}_{1}$ mode) which is available to be transferred into the output mode ${\hat{b}}_{\text{out}}$ .

**Figure 4:**
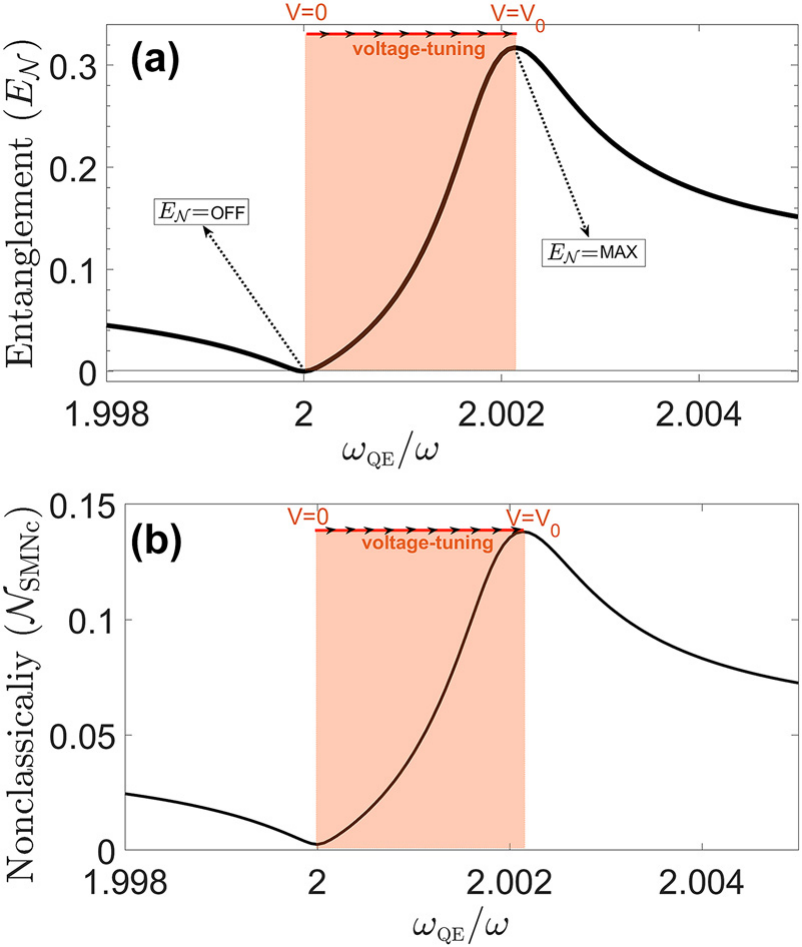
Generation of non-classicality and entanglement. (a) Degree of the entanglement (log-neg) of the two output fields *δa*_out_ and *δb*_out_ in Figure 1. Voltage-tuning of the QE(s) level-spacing between *ω*_QE_ = 2*ω* and *ω*_QE_ ≈ 2.002*ω* turns off and on the entanglement, respectively. Thus an undesired wave is not turned into non-classical. (b) Single-mode non-classicality $\left(N_{\text{SMNc}}\right)$ of the output mode ${\hat{b}}_{\text{out}}$ in units of log-neg [48, 64]. The right-going output can be used to generate entanglement via an integrated beam-splitter [44, 45, 47].

The two output modes of the cavity, ${\hat{a}}_{\text{out}}$ and ${\hat{b}}_{\text{out}}$ , are also entangled with each other. This is because the cavity mode ${\hat{c}}_{1}$ is coupled to the both output modes —please also note that ${\hat{a}}_{\text{out}}$ and ${\hat{b}}_{\text{out}}$ are related to the cavity mode ${\hat{c}}_{1}$ via input–output relations [54, 55, 59]. Due to the common interaction with the cavity mode (entanglement swap [60]) —and due to the non-classicality in ${\hat{c}}_{1}$ — the ${\hat{a}}_{\text{out}}$ and ${\hat{b}}_{\text{out}}$ modes get entangled, see Figure 4a.

### 2.2 Langevin equations

Time evolution of the operators can be determined using the Heisenberg equations of motion, e.g., ${\dot{\hat{a}}}_{1}=\left[{\hat{a}}_{1},\hat{H}\right]$ , as

(1) $${\dot{\hat{c}}}_{1}=-\left(\kappa_{1}+i\omega_{1}\right){\hat{c}}_{1}-ig_{1}^{*}{\hat{a}}_{1}+\varepsilon_{L}{\text{e}}^{-\text{i}\omega t},$$

(2) $${\dot{\hat{c}}}_{2}=-\left(\kappa_{2}+i\omega_{2}\right){\hat{c}}_{2}-ig_{2}^{*}{\hat{a}}_{2},$$

(3) $${\dot{\hat{a}}}_{1}=-\left(\gamma_{1}+i\Omega_{1}\right){\hat{a}}_{1}-ig_{1}{\hat{c}}_{1}-i2\chi^{\left(2\right)}{\hat{a}}_{1}^{†}{\hat{a}}_{2},$$

(4) $${\dot{\hat{a}}}_{2}=-\left(\gamma_{2}+i\Omega_{2}\right){\hat{a}}_{2}-ig_{2}{\hat{c}}_{2}-i\chi^{\left(2\right)}{\hat{a}}_{1}^{2}-if{\hat{\rho }}_{eg},$$

(5) $${\dot{\hat{\rho }}}_{eg}=-\left(\gamma_{eg}+i\omega_{\text{QE}}\right){\hat{\rho }}_{eg}+if{\hat{a}}_{2}\left({\hat{\rho }}_{ee}-{\hat{\rho }}_{gg}\right),$$

(6) $${\dot{\hat{\rho }}}_{ee}=-\gamma_{ee}{\hat{\rho }}_{ee}+i2f\left.\left({\hat{a}}_{2}^{†}{\hat{\rho }}_{eg}-H.c.\right)\right],$$

where *κ*_1,2_ and *γ*_1,2_ are the decay rates for the cavity and plasmon modes. *γ*_*ee*_, and *γ*_*eg*_ are the diagonal and off-diagonal decay rates of the QE(s). Please see Supplementary Material [61] for details.

### 2.3 Fano control

One can find the field amplitudes of the coupled cavity-MNS-QE(s) system examining the expectations of the operators, e.g., $\alpha_{1,2}=\left\langle {\hat{a}}_{1,2}\right\rangle$ . Here, |*α*_2_|^2^ gives the number of SH converted plasmons which governs the non-classicality of the system. The steady-state amplitude of the second harmonic plasmons [40, 62, 63]

(7) $$\alpha_{2}=\frac{i\chi^{\left(2\right)}}{\frac{|f{|}^{2}y}{i\left(\omega_{\text{QE}}-2\omega \right)+\gamma_{eg}}-\left[i\left(\Omega_{2}-2\omega \right)+\gamma_{2}\right]}\alpha_{1}^{2}$$

is governed by the interference taking place in the denominator $D\left(\omega \right)=\frac{|f{|}^{2}y}{i\left(\omega_{\text{QE}}-2\omega \right)+\gamma_{eg}}-\left[i\left(\Omega_{2}-2\omega \right)+\gamma_{2}\right]$ . When *ω*_QE_ = 2*ω*, the first term of D becomes |*f*|^2^*y*/*γ*_*eg*_ which turns out to be very large due to the QE’s small decay rate [26], e.g., *γ*_*eg*_ = 10^−6^*ω*. The typical values for MNS-QE coupling and population inversion are *f* = 0.1*ω* and *y* = *ρ*_*ee*_ − *ρ*_*gg*_ ∼ − 1. Thus, the first term of D becomes of order $∼10^{4}\omega$ while the second term of D is less than unity (1 × *ω*). This greatly suppresses the SHG which is depicted in Figure 2. We remark that without the presence of the QE, the SHG of the MNS is governed by the second term of D . The suppression is stronger when sharper resonance QE(s) are used.

In contrast, one can also enhance the SHG by preforming a cancellation in the denominator D . By tuning *ω*_QE_ accordingly, the off-resonant expression (Ω_2_ − 2*ω*) can be canceled by the imaginary part of the first term in D , see *ω*_QE_ ≈ 2.002*ω* in Figure 2. Therefore, tuning the level-spacing of the QE *ω*_QE_ about ∼meV one can continuously tune the SHG by 5-orders of magnitude and in particular turn on and off the non-classicality. We utilize this phenomenon as a voltage-controlled integrable quantum entanglement device.

### 2.4 Generation of non-classicality and entanglement

We calculate the non-classicality of the system using the standard (quantum noise) methods [59]. The quantum non-classicality features of a system is determined solely by the fluctuations [65] (noise, e.g., $\delta {\hat{a}}_{1}$ ) around the expectations values of the fields $\left(\alpha_{1}=\left\langle {\hat{a}}_{1}\right\rangle \right)$ , i.e., ${\hat{a}}_{1}=\alpha_{1}+\delta {\hat{a}}_{1}$ and ${\hat{c}}_{1}=\beta_{1}+\delta {\hat{c}}_{1}$ . The Langevin equations for the noise operators can be written as

(8a) $$\delta {\dot{\hat{c}}}_{1}=-\left[\right.\kappa_{1}+i\left(\omega_{1}-\omega \right)\delta {\hat{c}}_{1}-ig_{1}^{*}\delta {\hat{a}}_{1}+\delta {\hat{c}}_{in}^{\left(1\right)},$$

(8b) $$\delta {\dot{\hat{c}}}_{2}=-\left[\right.\kappa_{2}+i\left(\omega_{2}-2\omega \right)\delta {\hat{c}}_{2}-ig_{2}^{*}\delta {\hat{a}}_{2}+\delta {\hat{c}}_{in}^{\left(2\right)},$$

(8c) $$\begin{array}{ll} \delta {\dot{\hat{a}}}_{1} & =-\left[\Gamma_{1}+i\left(\Omega_{1}-\omega \right)\right]\delta {\hat{a}}_{1}-ig_{1}\delta {\hat{c}}_{1}\\ & \;-2i\chi^{\left(2\right)}\left(\alpha_{1}^{*}\delta {\hat{a}}_{2}+\alpha_{2}\delta {\hat{a}}_{1}\right), \end{array}$$

(8d) $$\begin{array}{ll} \delta {\dot{\hat{a}}}_{2} & =-\left[\Gamma_{2}+i\left(\Omega_{2}-2\omega \right)\right]\delta {\hat{a}}_{2}-ig_{2}\delta {\hat{c}}_{2}\\ & \;-i\chi^{\left(2\right)}\left(2\alpha_{1}\delta {\hat{a}}_{1}\right). \end{array}$$

Quantum optics experiments with MNSs [66], [67], [68], [69], [70] demonstrate that, intriguingly, plasmon excitations can preserve entanglement much longer times [66], [67], [68], [69, 71, 72] compared to their decay rates controlling field amplitudes. For this reason, empirically, we need to consider a smaller decoherence rate Γ_1,2_ for the noise operators $\delta {\hat{a}}_{1,2}$ . The experiments show that Γ_1,2_ for a gold MNS can be at most 10^11^ Hz [67] —the value we employ in our calculations. This is roughly calculated from an experiment where squeezed light is first transformed into plasmon oscillations and propagated over a gold metal stripe of thickness 2 mm, i.e., 1/Γ = 2 mm/*c* ∼ 10^−11^ s [73]. We present a detailed analysis of the losses responsible for the degrading of the entanglement/squeezing in the Supplementary Material [61]. (We also present our results with Γ_1,2_ = *γ*_1,2_ in the Supplementary Material (SM) [61] for completeness.) Here, we need to remark that in our device, metallic loss of non-classicality takes place only at the metal nanostructure (MNS), which is much smaller than cavity dimensions.

First, we calculate the entanglement between the reflected $\left({\hat{a}}_{\text{out}}\right)$ and transmitted $\left({\hat{b}}_{\text{out}}\right)$ modes, see Figure 1. We calculate the logarithmic-negativity $\left(E_{N}\right)$ [74] which is an entanglement measure for Gaussian states [75]. As we use the standard linearization method [59] for calculating the evolution of the noise operators, the fields stay Gaussian and $E_{N}$ can be employed as a measure. In Figure 4a, we observe that the log-neg can be continuously tuned between $E_{N}$ = 0 (off) and $E_{N}$ = 0.3 (on) when the level-spacing is adjusted between *ω*_QE_ = 2.000*ω* and *ω*_QE_ = 2.002*ω*, respectively.

Next, we also calculate the single-mode non-classicality $\left(N_{SMNc}\right)$ of the transmitted wave ${\hat{b}}_{\text{out}}$ . We employ the entanglement potential [64] as measure for the single-mode non-classicality. Only a non-classical single-mode state can create entanglement at the output of a BS [47]. Entanglement potential is the degree of entanglement a non-classical state creates at the BS output which can also be quantified in terms of log-neg. We remind that not all of the non-classicality of a mode could be converted into entanglement at the BS output. In Figure 4b, we observe that $N_{SMNc}$ can similarly be continuously tuned by several orders of magnitude via a ∼meV adjustment of the QE level-spacing [26, 30], [31], [32], [33]. We note that energy level-spacing modulations as large as ∼25 meV [30], or even larger ones [76, 77] are observed.

Therefore, one can use the (i) entanglement of two waves propagating in opposite directions or (ii) convert the non-classicality of the transmitted ${\hat{b}}_{\text{out}}$ mode into entanglement on the right hand side using an integrated BS. Such a modulation is important for generating the non-classicality when the desired wave is passing through the device, but turning it off for an unwanted wave. We note that the sample setup, Figure 1, can be placed even into a smaller cavity [78].

## 3 Possible experimental implementations of the active entanglement device

The abovementioned narrow-band and tunable Fano resonance-based device architecture promises for the electrically controllable switches that paves the way towards the active control of the non-classicality generation. Although the electrically tunable Fano resonance device has so far not been experimentally realized as a non-classicality switch, the suggested system of QE positioned at the hot spot of the MNS has experimentally been demonstrated to be applicable using various methodologies [79], [80], [81]. Lyamkina et al. demonstrated a light–matter coupling of single InAs quantum dots monolithically integrated into electromagnetic hot-spots of sub-wavelength sized metal nanoantennas [79]. The authors formed self-assembled InAs quantum dots (QDs) on a molecular beam epitaxy grown (001) GaAs substrates and lithographically formed the bow-tie antenna structures followed by an e-beam metallization and lift-off. The antennas are reported to enhance the emission intensity of single QD by 16 times and the fabricated structure allows intrinsic electrical connectivity allowing for the Stark tuning of the QEs.

Alternately, Santosh et al. demonstrated vacuum Rabi splitting by using the strong coupling of silver bow-tie plasmonic cavities loaded with semiconductor quantum dots [80]. Authors used interfacial capillary forces to drive commercially available CdSe/ZnS QDs into the lithographically patterned holes in the bowtie gaps. A strong coupling rate of 120 meV has been reported with a single QD system and, by using the fabricated structures, authors demonstrated the transparency dip in the spectral measurements due to the Rabi splitting. Jiang et al. reported an automatically located QE structure by plasmonic nanoantennas bypassing the accurate nanoscale alignment of the source at the plasmonic hotspot [81]. The authors have demonstrated that 11 nm diameter, single CdSe/ZnS QDs could be trapped achieving a trap stiffness of 0.6 (fN/nm)/mW yielding 7 times increased brightness, 2 times shortened lifetime.

The waveguide integration and CMOS compatibility is another experimental aspect that should be satisfied for the realization of entanglement devices yielding the production of IQCs. To that end, number of fabrication approaches has been reported that are compatible to the suggested model of entanglement (SHG) device. Hallett et al. [26] demonstrated the electrical control of resonant photon scattering from QDs that are embedded in a waveguide coupled photonic device to provide a switchable nonlinear response at the single photon level. Our proposed approach is compatible to be implemented as an embedding of QEs inside the bulk p-i-n or Schottky diode structure that could provide a fast frequency tuning of the QEs with the DC Stark effect.

More interestingly, integrating synthetic systems such as *π*-conjugated molecules and colloids between the metallic antenna structures can provide the quantum-confined Stark effect that can effectively tune the QE’s wavelength due to the intrinsic charge carrier confinement in three dimensions. Although the synthetic QDs suffer from the heterogeneity that can create undesirable temporal fluctuations on a single particle level [77], Muller et al. have demonstrated a controlled manipulation of the single particle wave function in semiconducting colloidal QDs by asymmetric growth of shell materials that yields the localization of charge carriers at specific distances from the core [76]. To that end, synthetic QEs could provide more scalable and low-cost SHG device fabrication routes compared to epitaxial grown structures.

Furthermore, electrical tunability of the non-classicality switch can be limited by the screening of the gate electric fields. To overcome that, Shibata et al. have demonstrated the use of a liquid gate electrical double layer (EDL) in gating of zero dimensional QDs allowing to tune the electronic states over a wide range that is not possible in solid state dielectric gate transistors [30]. The authors have found that the efficiency of EDL gating (350 meV/V) is 6 times higher than the back gating (60 meV/V) for the QDs yielding around 25 meV charge addition energy between *N* = 1 and 2. Although the ionic liquid gate technology has been demonstrated as a powerful tool to effectively shift the Fermi energy in solids [82], [83], [84], the fast cyclic switching, and the waveguide integration remained limited due to the involvement of a liquid state material that operates via the propagation of the ionic content. However, with the development of CMOS compatible thin film capacitors, effective gating technologies promise for the development of active SHG devices that are coupled within a waveguide structure.

The recent momentum in the van der Waals heterostructures based research has revealed the usage of defect states as QEs. Schwarz et al. [32] have demonstrated the electrically pumped sharp luminescence from individual defects in WSe2 that are sandwiched in a graphene/hBN/WSe2/hBN/graphene structure. Our active SHG device using metallic nanoantenna structure(MNS) could be formed by using 2D materials such that MNS/local defect/MNS is formed. To achieve integrated photonics technology, waveguide coupled structures could be realized following the reported integration methods of 2D materials [85], [86], [87].

*System Parameters* — It is worth noting that in our results, the parameters (e.g., resonances and damping) are scaled with the pump frequency *ω*. Here, *ω* is around optical frequencies. For instance, if we associate this value with the laser source having a wavelength (*λ* = 2*πc*/*ω*) of 1064 nm, the rest of the parameters can be assigned accordingly. This is discussed also in the SM [61] extensively. Since, we are interested in a Fano resonance, the interaction strength between the nanoparticle and the QE can be, for instance, about *f* ∼ 15 meV, which is smaller than the plasmonic dephasing rate, *γ*_*p*_ ∼ 0.1 eV. This ensures the weak-enough-coupling regime required for a Fano resonance [88]. These values can be realized in experiments by choosing mode-volumes of the photonic (can also be a plasmonic) cavity accordingly [89, 90]. As QE level-spacing is chosen around the ${\hat{a}}_{2}$ plasmon mode —for Fano interference to take place at the converted frequency— for such a system it comes out to be about *λ*_*eg*_ = 532 nm. For these parameters, a level-spacing of 0.002*ω*, see Figure 4, corresponds to a meV tuning of the level-spacing by voltage.

## 4 Summary and discussions

In summary, we introduce a micron-scale quantum entanglement device which can be integrated into (quantum) photonic circuits. The non-classicality can be supplied into the integrated quantum circuit on-demand by applying a voltage on the device. The extraordinary large modulation depth (10^5^) results from the suppression feature of the Fano resonance (in the nonlinear response). Moreover, the linear response of the device is not altered in the tuning [33, 91, 92]. Although we study the tuning of non-classicality on a second harmonic generation process here, the same method can be applied also on the voltage-tuning of other nonlinear processes. The details of our calculations following the standard methods can be found in the SM [61].

Finally, we would like to point out that use of valley photonic crystals (VPCs) [93] would greatly help the circuit design. The VPCs enable robust unidirectional flow of light which avoids reflection at the cavity input (i.e., ${\hat{a}}_{\text{out}}$ ). This not only yields better field/nonlinearity enhancements, but also makes the transmitted squeezed light ${\hat{b}}_{\text{out}}$ have similar amplitude with the input enabling easier homodyne detections in experiments.

## Supplementary Material

Supplementary Material Details
