# Supplementary material for: On-demand continuous-variable quantum entanglement source for integrated circuits
Source: Nanophotonics. 2023 Jan 18;12(2):229–37. doi: 10.1515/nanoph-2022-0555 (PMC11501490; doi:10.1515/nanoph-2022-0555)
Supplement: Supplementary file 1 — Supplementary Material Details [file j_nanoph-2022-0555_suppl_001.pdf]

SUPPLEMENTARY MATERIAL:
On-demand continuous-variable quantum entanglement source for integrated circuits

Mehmet Günay[†,1], Priyam Das[†,2], Emre Yuce[3], Emre Ozan Polat[4], Alpan Bek[3], and Mehmet Emre Tasgin[5]
[†] *Contributed equally*
[1] *Department of Nanoscience and Nanotechnology, Faculty of Arts and Science, Mehmet Akif Ersoy University, 15030 Burdur, Turkey*
[2] *Department of Physics, Bankura Sammilani College, Kenduadihi, Bankura, WB-722101, India*
[3] *Department of Physics, Middle East Technical University, 06100 Ankara, Turkey*
[4] *Faculty of Engineering and Natural Sciences, Kadir Has University, Cibali, Istanbul 34083, Turkey and*
[5] *Institute of Nuclear Sciences, Hacettepe University, 06800 Ankara, Turkey*

In this supplementary material, we present the details for the Hamiltonian of the coupled cavity-MNS-QE system, input-output formalism and calculations of the entanglement and single-mode nonclassicality. First, we demonstrate the input-output formalism for a single-mode cavity in Section I. In Section II, we give the detailed analysis of the coupled system and derive the equations of motion for both the classical fields and the associated quantum fluctuations. Next, we present the calculations of two-mode entanglement and the single-mode nonclassicality in Section III and derive the expectation values in Section IV.

I. INPUT-OUTPUT FORMALISM

In this section, we give the details of the input-output formalism that we used in the main text. Let us consider a single cavity mode ($\hat{a}$) interacting with a reservoir. An optical cavity is generally described by a Hamiltonian of the form [1],

$$H = H_{sys} + H_b + H_{int}, \tag{S1}$$

where H_{sys} is function of internal-mode operators only. $H_b = \sum_k \hbar\omega_k \hat{b}_k^\dagger \hat{b}_k$ is the free Hamiltonian of the bath, and, ve $H_{int} = \hbar \sum_k g_k(\hat{b}_k^\dagger \hat{a} + \hat{a}^\dagger \hat{b}_k)$ describes the interaction between bath and cavity field [1].

$$i\hbar\frac{d\hat{b}_k}{dt} = [b, H] = \hbar\omega_k\hat{b} + \hbar g_k\hat{a} \tag{S2}$$

The reservoir operator $\hat{b}_k$ can be assumed in the following form for $t > t_0$ [1]:

$$\hat{b}_k(t) = e^{-i\omega_k(t-t_0)}\hat{b}_k(t_0) - ig_k\int_{t_0}^{t} e^{-i\omega_k(t-t')}\hat{a}(t')dt'. \tag{S3}$$

Here the first term is the free evolution of the reservoir mode and the second term is the interaction between harmonic oscillator and reservoir. For a time $t_1 > t$, one can write the reservoir operator $\hat{b}_k$ as [1],

$$\hat{b}_k(t) = e^{-i\omega_k(t-t_1)}\hat{b}_k(t_1) + ig_k\int_{t}^{t_1} e^{-i\omega_k(t-t')}\hat{a}(t')dt'. \tag{S4}$$

Heisenberg equation of motion for harmonic oscillator operator $\hat{a}$ is:

$$i\hbar\frac{d\hat{a}}{dt} = [\hat{a}, \hat{H}_{sys}] + \hbar\sum_k g_k\hat{b}_k. \tag{S5}$$

Here $\hat{a}$ is the annihilation operator for the harmonic oscillator. By using Eq.(S3) the operator can be found for $t > t_0$ [1]:

$$\begin{aligned}\frac{d\hat{a}}{dt} &= -\frac{i}{\hbar}[a, H_{sys}] - i\sum_k g_k e^{-i\omega_k(t-t_0)}\hat{b}_k(t_0) \\ &- \sum_k g_k^2\int_{t_0}^{t} e^{-i\omega_k(t-t')}\hat{a}(t')dt'.\end{aligned} \tag{S6}$$

In Eq. (S6), the third term includes the following integral, $I = -\sum_k g_k^2 \int_{t_0}^t e^{-i\omega_k(t-t')}\hat{a}(t')dt'$. In the transformation from discrete to continuum, we use the following: $\sum_k \rightarrow 2(L/2\pi)^3 \int d^3k$. Therefore, the integral I can be simplified as,

$$\begin{aligned}I &= -\frac{L^3}{\pi^2c^3}\int_0^\infty d\omega_k\, \omega_k^2 g_k^2 \int_{t_0}^{t} e^{-i\omega_k(t-t')}\hat{a}(t')dt' \\ &= D(\omega)g^2(\omega)\int_{-\infty}^{\infty} d\omega_k \int_{t_0}^{t} e^{-i\omega_k(t-t')}\hat{a}(t')dt' \\ &= -\frac{\eta}{2}\hat{a}(t)\end{aligned} \tag{S7}$$

where $\eta = 2\pi D(\omega)g^2(\omega)$, with $D(\omega) = L^3\omega^2/(\pi^2c^3)$. Here c is the velocity of light, L is the length of the cavity. The annihilation operator for time $t_1 > t$ can be written as,

$$\begin{aligned}\frac{d\hat{a}}{dt} &= -\frac{i}{\hbar}[a, H_{sys}] - i\sum_k g_k e^{-i\omega_k(t-t_0)}\hat{b}_k(t_0) \\ &+ \sum_k g_k^2\int_{t}^{t_1} e^{-i\omega_k(t-t')}\hat{a}(t')dt'.\end{aligned} \tag{S8}$$

Now, using Eq.(S6), (S7) and (S8) and after some algebra, we finally obtained the following two equations in terms of the input and output field:

$$\begin{aligned}\frac{d\hat{a}}{dt} &= -\frac{i}{\hbar}[a, H_{sys}] - \frac{\eta}{2}\hat{a}(t) + g(\omega)\hat{a}_{in}(t) \\ &= -\frac{i}{\hbar}[a, H_{sys}] + \frac{\eta}{2}\hat{a}(t) - g(\omega)\hat{a}_{out}(t).\end{aligned} \tag{S9}$$

We evaluate the integrals and then using Eq.(S9), we find the input-output relations for two-sided cavity as:

$$\hat{a}_{out}(t) + \hat{a}_{in}(t) = 2\pi D(\omega) g(\omega) \hat{a}(t), \tag{S10}$$

which can be generalized to two cavity-mode.

II. HAMILTONIAN AND EQUATIONS OF MOTION

We consider a photonic crystal cavity, consisting of a quantum emitter(s) placed at the hotspot of a metal nanoparticle structure, as shown in Fig. 1 in the main text. Quantum emitter(s) can be a quantum dot or defect centers on a nanodiamond or 2D material. The dynamics of the system is as follows. The fundamental cavity mode ($\hat{c}_1$) is being continuously pumped ($\varepsilon_L^{(1)}$) with frequency ω, which excites the first-harmonic mode ($\hat{a}_1$) of the plasmonic structure. The generated plasmonic second-harmonic mode ($\hat{a}_2$) couples to both the second cavity mode ($\hat{c}_2$) and the quantum emitter with coupling strengths g_2 and f_2, respectively. The total Hamiltonian of this system can be given as $\hat{H} = \hat{H}_0 + \hat{H}_{\rm int} + \hat{H}_{\rm SH}$, where

$$\begin{aligned} \hat{H}_0 &= \hbar\omega_{eg}\hat{\rho}_{ee} + \sum_{i=1}^{2} \hbar(\omega_i \hat{a}_i^\dagger \hat{a}_i + \omega_{ci}\hat{c}_i^\dagger \hat{c}_i), && \text{(S11)} \\ \hat{H}_{\rm int} &= \sum_{i=1}^{2} \hbar(g_i \hat{a}_i^\dagger \hat{c}_i + f_i \hat{a}_i^\dagger \hat{\rho}_{ge} + h.c.), && \text{(S12)} \\ \hat{H}_{\rm SH} &= \hbar\chi^{(2)}(\hat{a}_2^\dagger \hat{a}_1 \hat{a}_1 + \hat{a}_1^\dagger \hat{a}_1^\dagger \hat{a}_2). && \text{(S13)} \end{aligned}$$

Here, ω_{eg} corresponds to the level-spacing of the quantum emitter, with $\hat{\rho}_{ee} = |e\rangle\langle e|$, $\hat{\rho}_{eg} = |e\rangle\langle g| = \hat{\rho}_{ge}^\dagger$. $|g\rangle$ ($|e\rangle$) represents the ground (excited) state of the quantum emitter. $\hat{H}_{\rm int}$ is the coupling between the cavity fields and plasmon modes of the metal nanoparticles, with the coupling strength g_i and the interaction between the plasmonic structure and the quantum emitter, with respective strengths f_1 and f_2. The Hamiltonian of the second harmonic generation is given by $\hat{H}_{\rm SH}$, where $\chi^{(2)}$ being the second order correlation, and it is proportional to the second harmonic susceptibility of the plasmonic structure. The first (second) harmonic mode $\hat{a}_1$ ($\hat{a}_2$) with frequency ω_1 (ω_2) of the plasmonic structure couples to the cavity mode $\hat{c}_1$ ($\hat{c}_2$).

To derive the equation of motions corresponds to the cavity field and plasmonic structure-quantum emitter hybrid system, we use the Heisenberg equation of motion: $i\hbar\dot{\hat{O}} = [\hat{O}, \hat{H}]$, and obtain

$$\begin{aligned} \dot{\hat{c}}_j &= -(\kappa_j + i\omega_{cj})\hat{c}_j - ig_j^*\hat{a}_j + \varepsilon_L^{(j)} e^{-i\omega_j t}, && \text{(S14)} \\ \dot{\hat{a}}_1 &= -(\gamma_1 + i\omega_1)\hat{a}_1 - ig_1\hat{c}_1 - i2\chi^{(2)}\hat{a}_1^\dagger \hat{a}_2 - if_1\hat{\rho}_{ge}, && \text{(S15)} \\ \dot{\hat{a}}_2 &= -(\gamma_2 + i\omega_2)\hat{a}_2 - ig_2\hat{c}_2 - i\chi^{(2)}\hat{a}_1^2 - if_2\hat{\rho}_{ge}, && \text{(S16)} \\ \dot{\hat{\rho}}_{ge} &= -(\gamma_{eg} + i\omega_{eg})\hat{\rho}_{ge} + i(f_1\hat{a}_1 + f_2\hat{a}_2)(\hat{\rho}_{ee} - \hat{\rho}_{gg}), && \text{(S17)} \\ \dot{\hat{\rho}}_{ee} &= -\gamma_{ee}\hat{\rho}_{ee} + i2[(f_1\hat{a}_1^\dagger + f_2\hat{a}_2^\dagger)\hat{\rho}_{ge} - H.c.)], && \text{(S18)} \end{aligned}$$

where, we have introduced the decay rates κ_j, γ_j, γ_{ee}, and γ_{eg} of the cavity, plasmon modes and diagonal and off-diagonal components of the quantum emitter, respectively. $\varepsilon_L^{(j)}$ is the pump source of the jth cavity mode with $j = 1, 2$ and $\varepsilon_L^{(2)} = 0$. When the level spacing of the quantum emitter is of the order of SH frequency, $\omega_{eg} \sim 2\omega$, its interaction with the first harmonic mode becomes off-resonant, which can be neglected (i.e., $f_1 = 0$). We confirm this through 3-dimensional simulation in our earlier work, which converges to our ansantz for the steady state [2].

In the linear case, we ignore the terms like $\delta\hat{a}^2$, $\delta\hat{a}_1\delta\hat{a}_2$, and only consider the terms, which are linear in fluctuations. Therefore, the equation of motions in the linear approximation becomes:

$$\begin{aligned} \delta\dot{\hat{c}}_j &= -(\zeta_j + i\Delta_{cj})\delta\hat{c}_j - ig_j^*\delta\hat{a}_j + \delta\hat{c}_{in}^{(j)}, && \text{(S19a)} \\ \delta\dot{\hat{a}}_1 &= -(\Gamma_1 + i\Delta_1)\delta\hat{a}_1 - ig_1\delta\hat{c}_1 \\ &\quad - 2i\chi^{(2)}(\alpha_{a1}^*\delta\hat{a}_2 + \alpha_{a2}\delta\hat{a}_1), && \text{(S19b)} \\ \delta\dot{\hat{a}}_2 &= -(\Gamma_2 + i\Delta_2)\delta\hat{a}_2 - ig_2\delta\hat{c}_2 \\ &\quad - i\chi^{(2)}(2\alpha_{a1}\delta\hat{a}_1). && \text{(S19c)} \end{aligned}$$

The presence of the term $i\chi^{(2)}2\alpha_1\delta\hat{a}_1$ in Eq. (S19c) creates squeezing in the first SPP mode, $\hat{a}_1$. This squeezing of $\hat{a}_1$, can induce an entanglement in the two output fields. To see this, let us first calculate the depletion associated with each fields:

$$\delta\hat{x}_{oi} = \frac{1}{\sqrt{2}}(\delta\hat{o}_i^\dagger + \delta\hat{o}_i); \quad \delta\hat{p}_{oi} = \frac{i}{\sqrt{2}}(\delta\hat{o}_i^\dagger - \delta\hat{o}_i) \tag{S20}$$

The depletions associated with these fields require the knowledge of the hermitian conjugate of these fields, which can be obtained by taking the hermitian conjugate of Eqs. (S19a-S19c) and desired the depletion in momentum-space operators can be obtained in the following matrix form,

$$\overrightarrow{\dot{u}} = A.\overrightarrow{u} + \overrightarrow{u_{in}}, \tag{S21}$$

where, $\overrightarrow{u} = [\delta x_{c1}\ \delta p_{c1}\ \delta x_{c2}\ \delta p_{c2}\ \delta x_{a1}\ \delta p_{a1}\ \delta x_{a2}\ \delta p_{a2}]^T$, and $\overrightarrow{u_{in}} = [\delta x_{in}^{(1)}\ \delta p_{in}^{(1)}\ \delta x_{in}^{(2)}\ \delta p_{in}^{(2)}\ 0\ 0\ 0\ 0]^T$. The matrix A can be constructed by using Eqs. (S19a-S19c) and Eq. (S20), which is going to be 8×8 matrix.

$$A=\begin{bmatrix} -\zeta_1 & \Delta_{c1} & 0 & 0 & -g_{1I} & g_{1R} & 0 & 0\\ -\Delta_{c1} & -\zeta_1 & 0 & 0 & -g_{1R} & -g_{1I} & 0 & 0\\ 0 & 0 & -\zeta_2 & \Delta_{c2} & 0 & 0 & -g_{2I} & g_{2R}\\ 0 & 0 & -\Delta_{c2} & -\zeta_2 & 0 & 0 & -g_{2R} & -g_{2I}\\ g_{1I} & g_{1R} & 0 & 0 & (-\Gamma_1+\beta_{2I}) & (\Delta_1-\beta_{2R}) & -\beta_{1I} & \beta_{1R}\\ -g_{1R} & g_{1I} & 0 & 0 & -(\Delta_1-\beta_{2R}) & -(\Gamma_1+\beta_{2I}) & -\beta_{1R} & -\beta_{1I}\\ 0 & 0 & g_{2I} & g_{2R} & \beta_{1I} & \beta_{1R} & -\Gamma_2 & \Delta_2\\ 0 & 0 & -g_{2R} & g_{2I} & -\beta_{1R} & \beta_{1I} & -\Delta_2 & -\Gamma_2 \end{bmatrix}$$

where g_{iR} (g_{iI}) is the real (complex) part of the interaction term g_i.

The formal solution of the Eq. (S21) can be calculated for the cavity fields as

$$\delta\hat{x}_{c1}(t) = \sum_{l=1}^{8}\left\{M_{1l}u_l(0)+\int_0^t \mathrm{d}sM_{1l}(t-s)u_{in,l}(s)\right\} \tag{S22}$$

$$\delta\hat{p}_{c1}(t) = \sum_{l=1}^{8}\left\{M_{2l}u_l(0)+\int_0^t \mathrm{d}sM_{2l}(t-s)u_{in,l}(s)\right\} \tag{S23}$$

$$\delta\hat{x}_{c2}(t) = \sum_{l=1}^{8}\left\{M_{3l}u_l(0)+\int_0^t \mathrm{d}sM_{3l}(t-s)u_{in,l}(s)\right\} \tag{S24}$$

$$\delta\hat{p}_{c2}(t) = \sum_{l=1}^{8}\left\{M_{4l}u_l(0)+\int_0^t \mathrm{d}sM_{4l}(t-s)u_{in,l}(s)\right\} \tag{S25}$$

where $M(t)=e^{At}$. As $t\to\infty$, the matrix $M(t)$ goes to zero, due to its exponential behavior. We would like to emphasize that the matrix elements can be obtained analytically by diagonalizing e^{At} as

$$M(t)=e^{At}=Pe^{A_dt}P^{-1}=Pe^{A_dt}Q, \tag{S26}$$

where $e^{A_dt}=Qe^{At}P$ is the diagonal form of matrix A and P is a tranformation matrix with $Q=P^{-1}$. Then, the elements of the matrix $M(t)$ can be given as

$$M_{ij}(t)=[e^{At}]_{ij} = \sum_{l=1}^{8}P_{il}Q_{lj}e^{\lambda_l t}. \tag{S27}$$

It is worth pointing out that since the input fields, as well as noises are all Gaussian, and since the equations of motion are linearized, the output fields will also have the Gaussian structures [3]. From the definition $\delta\hat{c}_i=(\delta\hat{x}_{ci}+i\delta\hat{p}_{ci})/\sqrt{2}$ one obtains

$$\delta\hat{c}_i(t)=\sum_{l=1}^{8}\left\{f_{il}(t)u_l+\int_0^t ds f_{il}(t-s)u_{in,l}(s)\right\}, \tag{S28}$$

where, $f_{1l}(t)=M_{1l}(t)+iM_{2l}(t)$ and $f_{2l}(t)=M_{3l}(t)+iM_{4l}(t)$. Subsequently, we assumed that $\delta c_{in}^{(i)}(s)=g_{lc}^{(i)}\delta\hat{a}_{in}^{(i)}+g_{rc}^{(i)}\delta\hat{b}_{in}^{(i)}$, where, $\delta\hat{a}_{in}^{(i)}$ refers to the modes of the vacuum acting from the left (l) hand side of the cavity, whereas $\delta\hat{b}_{in}^{(i)}$ corresponds to the modes acting from the right (r) hand side of the cavity. Hence, these two operators commute with each other. In the light of these definitions, elements of the input vector can be given as

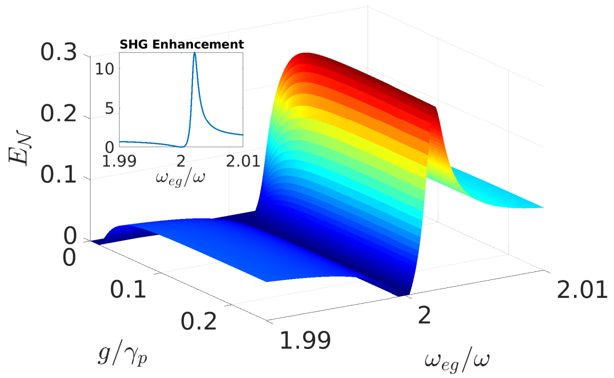

FIG. S1. The logarithmic negativity ($E_{\mathcal{N}}$) of the output fluctuations $\delta a_{\rm out}^{(1)}$ and $\delta b_{\rm out}^{(1)}$ as a function of quantum emitter level spacing (ω_{eg}) and cavity-plasmon coupling strength (g) for fixed plasmon-quantum emitter coupling ($f=\gamma_p/4$) and $\Gamma_p=10^{-3}\gamma_p$. In the inset, we demonstrate enhancement factor of SHG field with respect to ω_{eg}, obtained from Eq. (??). The rest of the parameters are given in text.

$$u_{in,1} = \frac{1}{\sqrt{2}}(\delta\hat{c}_{in}^{\dagger,(1)}(s)+\delta\hat{c}_{in}^{(1)}(s)), \tag{S29a}$$

$$u_{in,2} = \frac{i}{\sqrt{2}}(\delta\hat{c}_{in}^{\dagger,(1)}(s)-\delta\hat{c}_{in}^{(1)}(s)), \tag{S29b}$$

$$u_{in,3} = \frac{1}{\sqrt{2}}(\delta\hat{c}_{in}^{\dagger,(2)}(s)+\delta\hat{c}_{in}^{(2)}(s)), \tag{S29c}$$

$$u_{in,4} = \frac{i}{\sqrt{2}}(\delta\hat{c}_{in}^{\dagger,(2)}(s)-\delta\hat{c}_{in}^{(2)}(s)). \tag{S29d}$$

By using these definitions, one can obtain the expectation values of the output fields, which we present in the last Section.

III. TWO-MODE ENTANGLEMENT AND SINGLE-MODE NONCLASSICALITY

In this section, we give the detailed calculations for two-mode entanglement between the fluctuations of the two output pulses propagating in opposite directions, and

the single-mode nonclassicality (SMNc) of a fundamental cavity mode $\hat{c}_1$. Our calculations are based on logarithmic negativity, which is a commonly used quantity for witnessing the entanglement and the single-mode nonclassicality in Gaussian states [4–8]. Let us start with introducing correlation matrix for any two mode (mode-a and mode-b) [9], defined as

$$\mathcal{V} = \begin{pmatrix} W_{aa} & W_{ab} \\ W_{ba} & W_{bb} \end{pmatrix}, \tag{S30}$$

where the elements W_{ij} with $i, j : a, b$ are 2×2 matrix, given by

$$W_{ij} = \begin{pmatrix} W_{11}^{ij} & W_{12}^{ij} \\ W_{21}^{ij} & W_{22}^{ij} \end{pmatrix}, \tag{S31}$$

where

$$W_{11}^{ij} = \langle \delta\hat{x}_i \delta\hat{x}_j + \delta\hat{x}_j \delta\hat{x}_i \rangle/2 - \langle \delta\hat{x}_i \rangle \langle \delta\hat{x}_j \rangle \tag{S32}$$
$$W_{12}^{ij} = \langle \delta\hat{x}_i \delta\hat{p}_j + \delta\hat{p}_j \delta\hat{x}_i \rangle/2 - \langle \delta\hat{x}_i \rangle \langle \delta\hat{p}_j \rangle \tag{S33}$$
$$W_{21}^{ij} = \langle \delta\hat{p}_i \delta\hat{x}_j + \delta\hat{x}_j \delta\hat{p}_i \rangle/2 - \langle \delta\hat{p}_i \rangle \langle \delta\hat{x}_j \rangle \tag{S34}$$
$$W_{22}^{ij} = \langle \delta\hat{p}_i \delta\hat{p}_j + \delta\hat{p}_j \delta\hat{p}_i \rangle/2 - \langle \delta\hat{p}_i \rangle \langle \delta\hat{p}_j \rangle. \tag{S35}$$

Symplectic eigenvalues of the correlation matrix can be calculated as [10]

$$\nu_{\pm} = \frac{1}{\sqrt{2}} \left(\sigma(\mathcal{V}) \pm \{[\sigma(\mathcal{V})]^2 - 4\mathrm{det}(\mathcal{V})\}^{\frac{1}{2}} \right)^{\frac{1}{2}}. \tag{S36}$$

Here $\sigma(\mathcal{V}) = \det(W_{aa}) + \det(W_{bb}) - 2\det(W_{ab})$. A two-mode Gaussian state becomes entangled when $2\nu_- < 1$ by considering $\nu_- < \nu_+$, which is both a necessary and a sufficient criterion. Moreover, the logarithmic negativity

$$E_{\mathcal{N}} = \max\{0, -\frac{1}{2}\log_2(2\nu_-)\} \tag{S37}$$

is also commonly used as a quantifier for two-mode Gaussian systems. In Fig. S1, we demonstrate the logarithmic negativity of the output fluctuations $\delta a_{\rm out}^{(1)}$ and $\delta b_{\rm out}^{(1)}$ as a function of the level spacing of the quantum emitter. It is shown that when the quantum emitter becomes resonant (i.e., $\omega_{eg} = 2\omega$) the logarithmic negativity vanishes. It is the frequency that the nonlinear field is also suppressed (Fano resonance) [2]. Enhancement of the nonlinear conversion, on the other hand, leads to higher value for two-mode entanglement.

Here we scaled the decay rates with the pump frequency ω, and take $\gamma_1 = \gamma_2 = \gamma_p = 0.1\omega$, $\kappa_1 = \kappa_2 = 10^{-6}\omega$, $\Gamma_1 = \Gamma_2 = \Gamma_p = 10^{-4}\omega$, $\gamma_{eg} = 10^{-4}\omega$, the resonance values $\omega_{c1} = 10^{-5}\omega$, $\omega_1 = 10^{-1}\omega$, and the interactions $g = 0.4\gamma_p$, $f = \gamma_p/4$.

In the following, we consider the single-mode nonclassicality of the first cavity mode $\hat{c}_1$, which can also be measured in terms of the logarithmic negativity. Since two output modes $\hat{a}_{out}^{(1)}$ and $\hat{b}_{out}^{(1)}$ can be written in terms of the

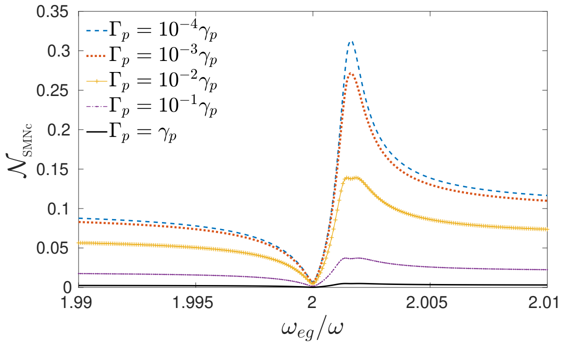

FIG. S2. The single-mode nonclassicality ($\mathcal{N}_{\rm SMNc}$) in the fluctuations of the fundamental cavity mode $\delta\hat{c}_1$ with respect to quantum emitter level spacing (ω_{eg}) for various decay rates of the fluctuations associated with the plasmon field (Γ_p) and for fixed interaction strength $g = 0.25\gamma_p$. The rest of the parameters are the same with the main text.

related cavity mode $\hat{c}_1$, one can calculate the nonclassicality of the $\delta\hat{c}_1$ by using linear beam-splitter (BS) operations [10, 11]. The single-mode nonclassicality measure $\mathcal{N}_{\rm SMNc}$ can be derived by maximazing $E_{\mathcal{N}}$ over the phase angle ϕ and tranmission coefficient t with constrained $r^2 + t^2 = 1$, where r is the reflection coefficient (detailed derivation can be found in Ref. [10]). The resulting correlation matrix of a single-mode for maximized values (i.e., $\phi = 0$ and $r = t = 1/\sqrt{2}$) can be obtained as

$$\mathcal{V} = \begin{pmatrix} B & C \\ C^T & B \end{pmatrix} \tag{S38}$$

where

$$B = \frac{1}{2} \begin{bmatrix} n + v\cos\theta + 1 & v\sin\theta \\ v\sin\theta & n - v\cos\theta + 1 \end{bmatrix}, \tag{S39}$$
$$C = \frac{1}{2} \begin{bmatrix} -n - v\cos\theta & -v\sin\theta \\ -v\sin\theta & -n + v\cos\theta \end{bmatrix}. \tag{S40}$$

Here $\langle \delta\hat{c}_1^\dagger \delta\hat{c}_1 \rangle = n$ and $\langle \delta\hat{c}_1 \delta\hat{c}_1 \rangle = ve^{i\theta}$ with v is real and positive.

Since the decay rates of the fluctuations can be much smaller in the plasmonic fields (i.e., $\gamma_p \gg \Gamma_p$) [12–14], the preservation of entanglement [15, 16] and squeezing [14, 17] can attain longer times. To see this, in Fig. S2, we demonstrate the role of the decay rates of the plasmon fluctuations on the single-mode nonclassicality. It can be seen from the figure that as the lifetime of the fluctuations increases, the single-mode nonclassicality takes higher values, which can be expected.

Losses in the device.— We underline that the device we propose forms from a silicon photonic cavity and a small metal nanostructure (MNS, plasmons) —the part which possesses a high damping ($\sim$ 10 fs). The device, the cavity, lies along a silicon photonic waveguide. That is, the only lossy part of the device is the 10s of nm sized MNS. Thus, of the loss of entanglement/squeezing

(nonclassicality) and the intensity are standard along the waveguide. The lossy part is the place where nonclassicality itself is generated via SHG process which is a small portion.

For instance, two experiments with squeezed plasmons, [17] and [14], show that nonclassicality (entanglement and squeezing) are preserved at worst for times $\Delta = L/c = 10^{-11}$ sec [17] and $\Delta = L/c = 10^{-10}$ sec [14], respectively. (The difference is not because there is discrepancy between two nonclassicality types. It is rather because the metal stripe in the experiment [17] is 1/10 length of the experiment [14].) We take the plasmon propagation speed as equal to c, which can actually be quite shorter. We also remark that squeezing and entanglement are totally equivalent nonclassicalities which can be converted into each other and a conservation-like relation holds [18–21]. In the calculations we use the worst-ever value $\Gamma_{1,2} = 10^{-4}\omega \sim 10^{11}$ Hz which corresponds to the former experiment [17].

IV. EXPECTATION VALUES

In this part, we give the details of the calculations of the expectation values of output fields. Let us start with

$$\begin{aligned}\langle \delta\hat{a}^{(1)}_{out}(t)\delta\hat{a}^{(1)}_{out}(t)\rangle &= \langle(\bar{g}^{(1)}_{lc}\delta\hat{c}_1(t) - \delta\hat{a}^{(1)}_{in}(t))(\bar{g}^{(1)}_{lc}\delta\hat{c}_1(t) - \delta\hat{a}^{(1)}_{in}(t))\rangle \\ &= \bar{g}^{(1)^2}_{lc}\langle\delta\hat{c}_1(t)\delta\hat{c}_1(t)\rangle - \bar{g}^{(1)}_{lc}\langle\delta\hat{a}^{(1)}_{in}(t)\delta\hat{c}_1(t)\rangle - \bar{g}^{(1)}_{lc}\langle\delta\hat{c}_1(t)\delta\hat{a}^{(1)}_{in}(t)\rangle + \langle\delta\hat{a}^{(1)}_{in}(t)\delta\hat{a}^{(1)}_{in}(t)\rangle \\ &= \bar{g}^{(1)^2}_{lc}\langle\delta\hat{c}_1(t)\delta\hat{c}_1(t)\rangle - \bar{g}^{(1)}_{lc}\langle\delta\hat{a}^{(1)}_{in}(t)\delta\hat{c}_1(t)\rangle\end{aligned} \quad \text{(S41)}$$

where, the last two terms in the Eq.(S41) become zero, since $\delta\hat{a}^{(1)}_{in}(t)$ operating on vacuum states gives zero. For the sake of notational convenience, we write $\delta\hat{c}_1(t) = \delta\hat{c}_1$ and so on. Let us first calculate expectation values:

$$\begin{aligned}\bar{g}^{(1)}_{lc}\langle\delta\hat{a}^{(1)}_{in}\delta\hat{c}_1\rangle &= \bar{g}^{(1)}_{lc}\sum_{l=1}^{8} f_l(t)\langle\delta\hat{a}^{(1)}_{in}u_l(0)\rangle + \bar{g}^{(1)}_{lc}\sum_{l=1}^{4}\int_0^t ds h_{1l}(t-s)\langle\delta\hat{a}^{(1)}_{in}u_{in,l}(s)\rangle \\ &= \bar{g}^{(1)}_{lc}\sum_{l=1}^{4}\int_0^t ds h_l(t-s)\langle\delta\hat{a}_{in}u_{in,l}(s)\rangle \\ &= \bar{g}^{(1)}_{lc}\sum_{l=1}^{4}\int_0^t ds h_l(t-s)\frac{1}{\sqrt{2}}[\langle\delta\hat{a}^{(1)}_{in}\delta\hat{c}^{(1),\dagger}_{in}\rangle + \langle\delta\hat{a}^{(1)}_{in}\delta\hat{c}^{(1)}_{in}\rangle] \\ &= \bar{g}^{(1)}_{lc}\sum_{l=1}^{4}\int_0^t ds h_l(t-s)\frac{1}{\sqrt{2}}g^{(1)}_{1c}\langle\delta\hat{a}^{(1)}_{in}(t)\delta\hat{a}^{(1),\dagger}_{in}(s)\rangle \\ \bar{g}^{(1)^2}_{lc}\langle\delta\hat{a}(t)\delta\hat{a}(t)\rangle &= \bar{g}^{(1)^2}_{lc}\sum_{m=1}^{8}\sum_{n=1}^{8} f_{1m}(t)f_{1n}(t)\langle u_{1m}(0)u_{1n}(0)\rangle \\ &+ \frac{1}{2}\bar{g}^{(1)^2}_{lc}(g^{(1)^2}_{1c} + g^{(1)^2}_{rc})2\pi D(\omega_{c1})\sum_{m=1}^{4}\sum_{n=1}^{4} V_{m,n}\int_0^t\int_0^{t'} ds ds' h_{1m}(t-s)h_{1n}(t'-s').\end{aligned} \quad \text{(S42)}$$

It will be easy to obtain the above expectation value using the definition $\hat{a}^{(i)}_{\rm in}(t) = -i\sum_k e^{-i\omega^{(i)}_k t}\hat{b}^{(i)}_k$, which is given by

$$\langle\delta\hat{a}^{(1)}_{in}(t)\delta\hat{a}^{(1),\dagger}_{in}(s)\rangle = 2\pi D(\omega_{c1})\delta(t-s).$$

and

$$\begin{aligned}\langle u_{in,1}(s)u_{in,1}(s')\rangle &= \frac{1}{2}\langle(\delta\hat{c}^{(1),\dagger}_{in} + \delta\hat{c}^{(1)}_{in})(\delta\hat{c}^{(1),\dagger}_{in} + \delta\hat{c}^{(1)}_{in})\rangle = \frac{1}{2}\langle\delta\hat{c}^{(1)}_{in}(s)\delta\hat{c}^{(1),\dagger}_{in}(s')\rangle \\ &= \frac{1}{2}[g^{(1)^2}_{lc}\langle\delta\hat{a}_{in}\delta\hat{a}^{\dagger}_{in}\rangle + g^{(1)^2}_{rc}\langle\delta\hat{b}_{in}\delta\hat{b}^{\dagger}_{in}\rangle = \frac{1}{2}(g^{(1)^2}_{lc} + g^{(1)^2}_{rc})2\pi D(\omega_{c1})\delta(s-s').\end{aligned} \quad \text{(S43)}$$

In a similar manner, one obtains the other expectation values: $\langle u_{in,i}(s)u_{in,j}(s')\rangle$ and inserting the results into the Eq.(S42), and after some algebra, we find

$$\begin{aligned}\langle\delta\hat{a}^{(1)}_{out}(t)\delta\hat{a}^{(1)}_{out}(t)\rangle &= \bar{g}^{(1)^2}_{lc}\sum_{m=1}^{8}\sum_{n=1}^{8}f_{1m}(t)f_{1n}(t)\langle u_{1m}(0)u_{1n}(0)\rangle \\ &+ \frac{1}{2}\bar{g}^{(1)^2}_{lc}(g^{(1)^2}_{lc}+g^{(1)^2}_{rc})2\pi D(\omega_{c1})\sum_{m=1}^{4}\sum_{n=1}^{4}V_{m,n}\int_0^t\int_0^{t'}dsds'h_{1m}(t-s)h_{1n}(t'-s') \\ &-\frac{1}{2}\bar{g}^{(1)}_{lc}g^{(1)}_{lc}2\pi D(\omega_{c1})[h_{11}(t)+h_{12}(t)]/\sqrt{2}. \end{aligned} \tag{S44}$$

Here, $V_{1,1}=V_{2,2}=V_{3,3}=V_{4,4}=1$, $V_{1,2}=-V_{2,1}=V_{3,4}=-V_{4,3}=i$ and rest are zero. Similarly, one can obtain the other expectation values of the output fields, which we present as follows:

$$\begin{aligned}\langle\delta\hat{a}^{(1)}_{out}(t)\delta\hat{b}^{(1)}_{out}(t)\rangle &= \bar{g}^{(1)}_{rc}\bar{g}^{(1)}_{lc}\sum_{m=1}^{8}\sum_{n=1}^{8}f_{1m}(t)f_{1n}(t)\langle u_{1m}(0)u_{1n}(0)\rangle \\ &+ \frac{1}{2}\bar{g}^{(1)}_{lc}\bar{g}^{(1)}_{rc}(g^{(1)^2}_{lc}+g^{(1)^2}_{rc})2\pi D(\omega_{c1})\sum_{m=1}^{4}\sum_{n=1}^{4}V_{m,n}\int_0^t\int_0^{t'}dsds'h_{1m}(t-s)h_{1n}(t'-s') \\ &-\frac{1}{2}\bar{g}^{(1)}_{lc}g^{(1)}_{lc}2\pi D(\omega_{c1})[h_{11}(t)+h_{12}(t)]/\sqrt{2}. \end{aligned} \tag{S45}$$

$$\begin{aligned}\langle\delta\hat{b}^{(1)}_{out}(t)\delta\hat{a}^{(1)}_{out}(t)\rangle &= \bar{g}_{lc}\bar{g}_{rc}\sum_{m=1}^{8}\sum_{n=1}^{8}f_{1m}(t)f_{1n}(t)\langle u_{1m}(0)u_{1n}(0)\rangle \\ &+\frac{1}{2}\bar{g}_{lc}\bar{g}_{rc}(g^2_{lc}+g^2_{rc})2\pi D(\omega_{c1})\sum_{m=1}^{4}\sum_{n=1}^{4}V_{m,n}\int_0^t\int_0^{t'}dsds'h_{1m}(t-s)h_{1n}(t'-s') \\ &-\frac{1}{2}\bar{g}_{rc}g_{rc}2\pi D(\omega_{c1})[h_{11}(t)+h_{12}(t)]/\sqrt{2}. \end{aligned} \tag{S46}$$

$$\begin{aligned}\langle\delta\hat{b}^{(1)}_{out}(t)\delta\hat{b}^{(1)}_{out}(t)\rangle &= \bar{g}^2_{rc}\sum_{m=1}^{8}\sum_{n=1}^{8}f_{1m}(t)f_{1n}(t)\langle u_{1m}(0)u_{1n}(0)\rangle \\ &+\frac{1}{2}\bar{g}^2_{rc}(g^2_{lc}+g^2_{rc})2\pi D(\omega_{c1})\sum_{m=1}^{4}\sum_{n=1}^{4}V_{m,n}\int_0^t\int_0^{t'}dsds'h_{1m}(t-s)h_{1n}(t'-s') \\ &-\frac{1}{2}\bar{g}_{rc}g_{rc}2\pi D(\omega_{c1})[h_{11}(t)+h_{12}(t)]/\sqrt{2}. \end{aligned} \tag{S47}$$

Following the similar approach, one can obtain the other correlation functions.

[1] M. O. Scully and M. S. Zubairy, Quantum optics, Cambridge Univ. Press (1997).

[2] M. Günay, Z. Artvin, A. Bek, and M. E. Tasgin, Controlling steady-state second harmonic signal via linear and nonlinear Fano resonances, Journal of Modern Optics **67**, 26 (2020).

[3] C. Genes, A. Mari, P. Tombesi, and D. Vitali, Robust entanglement of a micromechanical resonator with output optical fields, Physical Review A **78**, 032316 (2008).

[4] G. Adesso, A. Serafini, and F. Illuminati, Extremal entanglement and mixedness in continuous variable systems, Phys. Rev. A **70**, 022318 (2004).

[5] K. Życzkowski, P. Horodecki, A. Sanpera, and M. Lewenstein, Volume of the set of separable states, Phys. Rev. A **58**, 883 (1998).

[6] G. Vidal and R. F. Werner, Computable measure of entanglement, Phys. Rev. A **65**, 032314 (2002).

[7] M. B. Plenio, Logarithmic negativity: A full entanglement monotone that is not convex, Phys. Rev. Lett. **95**, 090503 (2005).

[8] S. Tserkis and T. C. Ralph, Quantifying entanglement in two-mode gaussian states, Phys. Rev. A **96**, 062338 (2017).

[9] M. E. Tasgin, Anatomy of entanglement and nonclassicality criteria, arXiv preprint arXiv:1901.04045 (2019).

[10] M. E. Tasgin, Measuring nonclassicality of single-mode

systems, Journal of Physics B: Atomic, Molecular and Optical Physics **53**, 175501 (2020).
[11] M. Hillery and M. S. Zubairy, Entanglement conditions for two-mode states: Applications, Phys. Rev. A **74**, 032333 (2006).
[12] S. Varró, N. Kroó, D. Oszetzky, A. Nagy, and A. Czitrovszky, Hanbury brown–twiss type correlations with surface plasmon light, Journal of Modern Optics **58**, 2049 (2011).
[13] G. Di Martino, Y. Sonnefraud, S. Kéna-Cohen, M. Tame, S. K. Ozdemir, M. Kim, and S. A. Maier, Quantum statistics of surface plasmon polaritons in metallic stripe waveguides, Nano Letters **12**, 2504 (2012).
[14] S. Fasel, M. Halder, N. Gisin, and H. Zbinden, Quantum superposition and entanglement of mesoscopic plasmons, New Journal of Physics **8**, 13 (2006).
[15] E. Altewischer, M. Van Exter, and J. Woerdman, Plasmon-assisted transmission of entangled photons, Nature **418**, 304 (2002).
[16] B. Lawrie, P. Evans, and R. Pooser, Extraordinary optical transmission of multimode quantum correlations via localized surface plasmons, Physical Review Letters **110**, 156802 (2013).
[17] A. Huck, S. Smolka, P. Lodahl, A. S. Sørensen, A. Boltasseva, J. Janousek, and U. L. Andersen, Demonstration of quadrature-squeezed surface plasmons in a gold waveguide, Physical Review Letters **102**, 246802 (2009).
[18] M. E. Tasgin, M. Gunay, and M. S. Zubairy, Nonclassicality and entanglement for wave packets, Phys. Rev. A **101**, 062316 (2020).
[19] I. I. Arkhipov, J. Peřina Jr, J. Svozilík, and A. Miranowicz, Nonclassicality invariant of general two-mode gaussian states, Scientific reports **6**, 26523 (2016).
[20] I. I. Arkhipov, J. Peřina Jr, J. Peřina, and A. Miranowicz, Interplay of nonclassicality and entanglement of two-mode gaussian fields generated in optical parametric processes, Physical Review A **94**, 013807 (2016).
[21] A. Černoch, K. Bartkiewicz, K. Lemr, and J. Soubusta, Experimental tests of coherence and entanglement conservation under unitary evolutions, Physical Review A **97**, 042305 (2018).
